# Supplementary material for: Regulatory Network Analysis in Estradiol-Treated Human Endothelial Cells
Source: Int J Mol Sci. 2021 Jul 30;22(15):8193. doi: 10.3390/ijms22158193 (PMC8348965; doi:10.3390/ijms22158193)
Supplement: Supplementary file 1 [file ijms-22-08193-s001.zip › Suppl. Table S3.pdf]

miRNA	Fold Change	Target	Fold Change
miR-30b-5p	2.02	JUN	-1.58
miR-25-5p	1.69	RREB1	-1.48
miR-6808-5p	1.65	CBX5	-2.86
miR-6808-5p	1.65	PPARA	-1.25
miR-6808-5p	1.65	ATF1	-1.29
miR-1296-5p	1.39	PLAG1	-1.35
miR-26a-5p	1.21	PLAG1	-1.35
miR-4524b-5p	1.15	PLAG1	-1.35
miR-512-3p	1.11	PPARA	-1.25
miR-92a-3p	1.08	NFIA	-1.52
miR-526b-3p	-1.15	STAT3	1.39
miR-4739	-1.15	THRA	1.27
miR-3936	-1.19	THRA	1.27
miR-1224-3p	-1.21	VDR	1.13
miR-4685-5p	-1.28	VDR	1.13
miR-4685-5p	-1.28	TEAD2	1.44
miR-4685-5p	-1.28	REPIN1	1.33
