# Supplementary material for: Regulatory Network Analysis in Estradiol-Treated Human Endothelial Cells
Source: Int J Mol Sci. 2021 Jul 30;22(15):8193. doi: 10.3390/ijms22158193 (PMC8348965; doi:10.3390/ijms22158193)
Supplement: Supplementary file 1 [file ijms-22-08193-s001.zip › Suppl. Table S4.pdf]

Transcription Factor	Target Genes	pvalue	Fold Change
**JUN_GENES**	EPHX1	0.0162	2.72
	ARID1A	0.0223	2.41
	LTBP3	0.0070	2.35
	ZYX	0.0032	2.21
	CORO1B	0.0393	2.16
	TGFB1	0.0301	2.09
	DPYSL4	0.0499	2.06
	PTOV1	0.0211	2.04
	C16orf58	0.0308	2.04
	CDC42EP5	0.0176	2.04
	EML3	0.0479	2.02
	DGKZ	0.0500	2.02
	SLC16A3	0.0294	2.00
	BOK	0.0167	2.00
	TLE2	0.0109	1.99
	TLN1	0.0037	1.96
	LMNA	0.0057	1.96
	MYL9	0.0053	1.94
	TMEM132A	0.0072	1.89
	TRIP6	0.0070	1.86
	PLEKHM2	0.0175	1.84
	ZC3H3	0.0476	1.82
	CPSF1	0.0366	1.82
	DMPK	0.0100	1.79
	PLEC	0.0073	1.79
	PLOD3	0.0052	1.78
	DOCK6	0.0214	1.76
	HGSNAT	0.0184	1.76
	COTL1	0.0246	1.74
	RAC2	0.0104	1.72
	CABIN1	0.0224	1.71
	GAS6	0.0243	1.71
	TMEM39B	0.0226	1.71
	SLC6A8	0.0150	1.70
	SMARCB1	0.0406	1.70
	PFN1	0.0154	1.69
	HDAC7	0.0140	1.68
	ASMTL	0.0059	1.68
	FAM69B	0.0085	1.68
	CYC1	0.0200	1.67
	FCGRT	0.0135	1.67
	CX3CL1	0.0445	1.66
	NBEAL2	0.0152	1.65
	MARCKSL1	0.0454	1.65

DOLK	0.0307	1.64
ERBB2	0.0481	1.63
MFSD10	0.0248	1.63
NADSYN1	0.0448	1.63
RAB1B	0.0386	1.62
MAP7D1	0.0133	1.61
FSCN1	0.0037	1.60
TNIP1	0.0338	1.60
C19orf43	0.0240	1.60
DTX3	0.0104	1.58
EEFSEC	0.0182	1.58
NDUFB11	0.0260	1.58
SLC2A6	0.0311	1.57
ZZEF1	0.0452	1.57
HMGA1	0.0167	1.57
FKBP8	0.0314	1.57
PLXNB3	0.0228	1.56
ALDOA	0.0162	1.55
SIGMAR1	0.0036	1.55
GBGT1	0.0296	1.55
MAP4	0.0431	1.54
ZNF513	0.0446	1.54
TUBB3	0.0000	1.53
ELF4	0.0121	1.53
ADD1	0.0044	1.52
KHNYN	0.0117	1.52
SYVN1	0.0310	1.52
PRKCDBP	0.0265	1.51
DYNC1H1	0.0228	1.51
AIF1L	0.0380	1.50
MFAP2	0.0437	1.50
STAT6	0.0036	1.49
HLA-G	0.0304	1.49
PSMG3	0.0443	1.48
PIM3	0.0203	1.48
S100A6	0.0309	1.48
LDB1	0.0421	1.48
CDC25B	0.0189	1.47
UBAP2L	0.0333	1.47
GADD45B	0.0118	1.46
TMEM8A	0.0088	1.45
ATG13	0.0458	1.45
TTYH3	0.0379	1.44
BCAR1	0.0141	1.44
NCAPH2	0.0477	1.44
NOTCH4	0.0002	1.43
PI4KB	0.0356	1.43

FAM127A	0.0206	1.43
KRT18	0.0384	1.42
CCDC86	0.0428	1.42
HYOU1	0.0187	1.42
AARS	0.0180	1.42
HK1	0.0365	1.41
COL6A1	0.0310	1.41
EHD2	0.0349	1.39
MDC1	0.0086	1.39
DCTN2	0.0288	1.38
VPS53	0.0248	1.37
INO80B	0.0177	1.37
ABL2	0.0475	1.37
SH3TC1	0.0077	1.36
EPN2	0.0391	1.35
COMT	0.0380	1.35
ICAM2	0.0248	1.35
IFITM2	0.0224	1.35
HPS1	0.0460	1.34
SVEP1	0.0003	1.34
RABGAP1	0.0287	1.34
KLC1	0.0378	1.34
AP2S1	0.0043	1.34
GTF2IRD1	0.0087	1.34
LSM11	0.0464	1.33
CABLES1	0.0027	1.33
REPIN1	0.0038	1.33
KDELR1	0.0058	1.33
PRMT2	0.0477	1.33
WWTR1	0.0286	1.33
PLAU	0.0025	1.32
TBCB	0.0419	1.32
CLN6	0.0030	1.32
CLCN7	0.0460	1.31
HLA-E	0.0031	1.31
PSMC4	0.0223	1.31
KIAA0319L	0.0053	1.31
SLC6A17	0.0097	1.31
XKR8	0.0072	1.30
NMT1	0.0340	1.30
SLC25A22	0.0306	1.29
GIT1	0.0338	1.29
GPAA1	0.0460	1.29
RAPGEF1	0.0079	1.28
BACE2	0.0069	1.28
PPP1R12C	0.0381	1.28
SLC1A5	0.0427	1.28

HIPK1	0.0290	1.27
ENO1	0.0086	1.27
ZNFX1	0.0397	1.27
NDUFC1	0.0037	1.27
SLC41A3	0.0462	1.26
SQSTM1	0.0138	1.26
BTNL9	0.0490	1.26
CYB5R3	0.0141	1.26
ERP29	0.0048	1.26
LCNL1	0.0241	1.26
ENPP1	0.0103	1.26
MICAL2	0.0440	1.25
SEC61A1	0.0067	1.25
ATAD3A	0.0306	1.25
ABCC10	0.0349	1.25
CA12	0.0383	1.24
PER1	0.0355	1.23
PDIA5	0.0018	1.23
FNDC5	0.0219	1.23
DNASE1	0.0326	1.23
MMRN2	0.0133	1.23
PEX5	0.0452	1.22
PTMS	0.0452	1.21
DCTN3	0.0490	1.21
TCP11L2	0.0249	1.20
JDP2	0.0369	1.19
MAPK8	0.0134	1.19
GAB2	0.0212	1.19
PRR5	0.0420	1.19
NDUFAB1	0.0161	1.18
CPXM1	0.0187	1.18
EPHA6	0.0237	1.18
HTR3A	0.0164	1.18
ZNF154	0.0411	1.17
DNAH1	0.0434	1.17
SNX18	0.0039	1.17
DERL3	0.0352	1.17
PNCK	0.0497	1.16
AQP4	0.0067	1.16
KIF19	0.0013	1.16
LY6K	0.0199	1.16
PRSS37	0.0353	1.15
PHPT1	0.0256	1.15
AGER	0.0384	1.15
PPP2R5B	0.0050	1.15
GLI3	0.0257	1.15
GRSF1	0.0074	1.14

RAET1E	0.0063	1.14
SLC35C2	0.0299	1.14
PCCA	0.0163	1.14
ZFP91	0.0108	1.14
HLA-DOB	0.0228	1.13
MLX	0.0463	1.12
MCTP2	0.0333	1.12
TLK1	0.0018	1.11
GFRA1	0.0026	1.10
WDR49	0.0376	1.10
MAP9	0.0365	1.10
BHLHE40	0.0216	1.09
CHST5	0.0408	1.08
PIPOX	0.0048	1.06
FANCC	0.0045	-1.05
VDAC2	0.0465	-1.07
PARK7	0.0308	-1.07
SFTA3	0.0143	-1.08
PTPRO	0.0176	-1.08
DCN	0.0422	-1.08
SLAMF9	0.0315	-1.10
SGCB	0.0113	-1.10
FZD1	0.0063	-1.10
HPGD	0.0201	-1.11
BLID	0.0391	-1.11
CASC5	0.0055	-1.11
SNTB1	0.0400	-1.11
EPHA3	0.0311	-1.12
USP14	0.0196	-1.12
SYT3	0.0493	-1.12
RAP1B	0.0008	-1.13
CASP2	0.0333	-1.13
CLCA4	0.0151	-1.14
DUSP13	0.0224	-1.14
YIPF4	0.0239	-1.14
PDZD3	0.0478	-1.14
CYP2C9	0.0331	-1.15
KIAA1919	0.0464	-1.15
ATP2B2	0.0021	-1.16
IQCK	0.0407	-1.17
IL27	0.0088	-1.17
C11orf57	0.0218	-1.17
HOPX	0.0278	-1.17
CLDN11	0.0077	-1.17
KCNJ16	0.0461	-1.18
NPM1	0.0115	-1.18
CA5B	0.0163	-1.18

LYSMD1	0.0146	-1.18
AREG	0.0010	-1.18
PCMT1	0.0221	-1.19
BLVRA	0.0106	-1.19
AKTIP	0.0403	-1.20
SLC24A4	0.0390	-1.20
CFLAR	0.0021	-1.21
AHI1	0.0297	-1.21
PCDHA9	0.0107	-1.22
ZNF445	0.0115	-1.22
SPTA1	0.0385	-1.22
ZMYM2	0.0098	-1.22
LIMS3	0.0075	-1.22
BTBD9	0.0266	-1.22
ACLY	0.0331	-1.22
ZNF780B	0.0066	-1.22
SETD6	0.0390	-1.23
CMIP	0.0350	-1.23
HEATR5B	0.0043	-1.23
PARVG	0.0229	-1.24
RAB8B	0.0341	-1.24
TDRD3	0.0085	-1.24
CNIH4	0.0151	-1.24
DENND4C	0.0382	-1.25
RPL15	0.0474	-1.25
PAPSS2	0.0394	-1.26
COPS8	0.0249	-1.26
TPD52L3	0.0310	-1.26
UQCRC2	0.0421	-1.26
SH3BP5	0.0116	-1.26
TM9SF3	0.0421	-1.27
CARD14	0.0075	-1.27
NMD3	0.0121	-1.28
CBX3	0.0005	-1.28
ELL3	0.0185	-1.28
ZNF175	0.0163	-1.29
NR2C1	0.0099	-1.29
RPL23	0.0366	-1.29
ANKRD26	0.0379	-1.29
PTPRB	0.0486	-1.29
UBE3A	0.0300	-1.29
CPM	0.0153	-1.30
ADK	0.0082	-1.30
VPS4B	0.0457	-1.31
RFTN2	0.0232	-1.31
MED24	0.0218	-1.31
AP1G1	0.0120	-1.32

	DDX3X	0.0261	-1.32
	SARNP	0.0465	-1.33
	NCOA3	0.0110	-1.33
	SLMAP	0.0198	-1.33
	TTC13	0.0488	-1.33
	ORC4	0.0141	-1.33
	CMKLR1	0.0199	-1.33
	MYO10	0.0091	-1.34
	KPNA4	0.0222	-1.34
	PPP2R5E	0.0267	-1.34
	TCF20	0.0138	-1.36
	MRVI1	0.0477	-1.36
	CCNC	0.0369	-1.37
	CSNK1G3	0.0222	-1.37
	DDR2	0.0366	-1.37
	USP53	0.0278	-1.37
	RBM39	0.0239	-1.38
	IFRD1	0.0420	-1.38
	GFM1	0.0204	-1.40
	ZNF10	0.0135	-1.40
	HELZ	0.0259	-1.41
	ZBTB20	0.0217	-1.41
	LRRCC1	0.0196	-1.41
	KDM4C	0.0361	-1.43
	ZNF587	0.0255	-1.46
	BNC2	0.0384	-1.47
	CRBN	0.0328	-1.48
	TMLHE	0.0202	-1.48
	DMXL1	0.0127	-1.49
	DNAJC1	0.0022	-1.50
	SBDS	0.0462	-1.50
	LRP2BP	0.0490	-1.51
	NFIA	0.0361	-1.52
	SPAG16	0.0272	-1.58
	RRP15	0.0424	-1.59
	CARD8	0.0061	-1.59
	SERPINB9	0.0221	-1.61
	CHMP5	0.0438	-1.64
	ATXN1	0.0332	-1.65
	CDC14B	0.0270	-1.65
	ERGIC2	0.0002	-1.70
	TAF9B	0.0271	-1.74
	GNAS	0.0198	-1.77
	TM2D1	0.0362	-1.84
	FOSL2	0.0106	-1.93
	AKAP2	0.0314	-1.96
	RASSF3	0.0462	-2.18

	NAA15	0.0345	-2.35
	ERAP1	0.0390	-2.83
	ARGLU1	0.0016	-2.83
	OGT	0.0258	-2.86
	TNRC6B	0.0118	-2.90
**RREB1_GENES**	VARS	0.0170	2.73
	PRRC2A	0.0398	1.81
	FLOT1	0.0103	1.81
	EHMT2	0.0485	1.57
	DDX39B	0.0177	1.50
	HLA-E	0.0031	1.31
**CBX5_GENES**	C1GALT1C1	0.0403	-1.36
	GTF2A1	0.0291	-1.21
	GTPBP10	0.0329	-1.31
	OR2F1	0.0086	1.08
	RPS27L	0.0181	-2.36
**PPARA_GENES**	GPR56	0.0364	1.83
	DALRD3	0.0259	1.65
	ACE	0.0143	1.60
	BCAR1	0.0141	1.44
	PTMS	0.0452	1.21
	CSPG4	0.0022	1.20
	UBL5	0.0462	1.14
	RARA	0.0020	1.13
	ANKRD9	0.0093	1.11
	RPS3A	0.0452	-1.08
	BHMT2	0.0423	-1.14
	PKP4	0.0290	-1.16
	NR2F6	0.0376	-1.16
	BAZ1B	0.0383	-1.22
	MARK3	0.0076	-1.24
	LDLRAD3	0.0403	-1.30
	PTPRE	0.0279	-1.32
	COG5	0.0164	-1.32
	C2orf27A	0.0284	-1.33
	TCF20	0.0138	-1.36
	KIAA0232	0.0058	-1.38
	KIAA1731	0.0107	-1.56
	DICER1	0.0080	-1.78
	JRKL	0.0096	-2.00
	PPP3CA	0.0015	-2.17
**ATF1_GENES**	PRRC2A	0.0397962	1.81
	COTL1	0.0246135	1.74
	YPEL3	0.0122296	1.69
	HES4	0.0496334	1.57

	PI4KB	0.0356287	1.43
	HK1	0.0364752	1.41
	SLC9A1	0.0129621	1.41
	TYK2	0.0241114	1.40
	SREBF2	0.0493517	1.40
	ZFAND2B	0.0496484	1.39
	CRELD1	0.0213071	1.37
	MPV17L2	0.000250357	1.37
	RNPS1	0.0178513	1.24
	TMUB2	0.031506	1.24
	C6orf132	0.0320494	1.20
	ADPRHL1	0.0105337	1.16
	FAM53C	0.0319929	1.14
	USP22	0.00248997	1.13
	BRF1	0.041197	1.12
	HSPA12A	0.0443255	1.11
	EAF2	0.0334967	1.09
	MCM10	0.0229955	-1.04
	PTPRO	0.0176464	-1.08
	NACA	0.0432938	-1.08
	RPL24	0.0328647	-1.17
	MURC	0.021704	-1.17
	ARL5B	0.0326243	-1.23
	KPNA4	0.0221878	-1.34
	UBE2N	0.0244434	-1.38
	ARMCX3	0.0300292	-1.47
	DNAJA2	0.0113841	-1.49
	UBE2B	0.0226948	-1.52
	JUN	0.0146453	-1.58
	RBBP6	0.0247192	-1.60
	KPNA5	0.045299	-1.60
	TPR	0.0299332	-1.77
**PLAG1_GENES**	DDX39B	0.0177	1.50
	EHMT2	0.0485	1.57
	FLOT1	0.0103	1.81
	HLA-E	0.0031	1.31
	PRRC2A	0.0398	1.81
	VARS	0.0170	2.73
**TEAD2_GENES**	RFC5	0.0035	-3.37
	SPTBN1	0.0057	-2.95
	RHEB	0.0450	-2.93
	OGT	0.0258	-2.86
	HDGFRP3	0.0032	-2.77
	COCH	0.0257	-2.48
	RPL27A	0.0174	-2.47

	UBE2Z	0.0278	-1.96
	TMEFF2	0.0081	-1.96
	TRIM13	0.0370	-1.92
	NFKBIZ	0.0147	-1.79
	SLC12A2	0.0025	-1.73
	RPL10	0.0486	-1.66
	ATP11A	0.0246	-1.62
	APLP2	0.0273	-1.60
	JUN	0.0146	-1.58
	KIAA1731	0.0107	-1.56
	DCUN1D1	0.0120	-1.48
	RREB1	0.0210	-1.48
	SS18L1	0.0365	-1.47
	SEMA6D	0.0131	-1.46
	CKS2	0.0329	-1.44
	NFX1	0.0078	-1.44
	MECOM	0.0435	-1.42
	QKI	0.0201	-1.41
	RIMKLB	0.0309	-1.38
	IFRD1	0.0420	-1.38
	MSI2	0.0493	-1.35
	EPS15L1	0.0312	-1.35
	ORC4	0.0141	-1.33
	ING2	0.0183	-1.32
	FBXO11	0.0423	-1.31
	CNOT6L	0.0471	-1.30
	CD58	0.0421	-1.30
	SMAD2	0.0396	-1.28
	PPP2R5C	0.0246	-1.28
	CBX3	0.0005	-1.28
	NMD3	0.0121	-1.28
	SOCS5	0.0197	-1.28
	CARD14	0.0075	-1.27
	JPH3	0.0175	-1.27
	RFPL2	0.0465	-1.27
	ADRBK2	0.0393	-1.26
	PER3	0.0479	-1.26
	CHCHD7	0.0473	-1.25
	AHCTF1	0.0483	-1.25
	ZNF345	0.0332	-1.24
	DEK	0.0264	-1.24
	RAB1A	0.0430	-1.22
	DPP6	0.0283	-1.22
	TBC1D24	0.0025	-1.21
	FBXL13	0.0039	-1.20

	OCLM	0.0034	-1.19
	KDELC1	0.0239	-1.19
	TBXA2R	0.0099	-1.18
	HNRNPL	0.0233	-1.18
	MED19	0.0406	-1.17
	ANO5	0.0400	-1.17
	NPAS3	0.0037	-1.16
	RASGRF1	0.0398	-1.16
	ATP2B2	0.0021	-1.16
	NR2F6	0.0376	-1.16
	GPBP1	0.0459	-1.15
	CDK5R1	0.0138	-1.14
	ANXA2	0.0310	-1.11
	C6orf223	0.0392	-1.09
	VSX1	0.0209	-1.08
	KCMF1	0.0113	-1.06
	IRS1	0.0299	-1.05
	PCSK4	0.0035	1.06
	CDHR5	0.0418	1.10
	RPS28	0.0117	1.10
	MRFAP1	0.0489	1.11
	CAMK2N2	0.0234	1.12
	H3F3A	0.0051	1.13
	MYL5	0.0379	1.13
	ZFP91	0.0108	1.14
	ATRNL1	0.0271	1.14
	TRMT61A	0.0427	1.14
	GLI3	0.0257	1.15
	COL8A2	0.0060	1.15
	EPHB2	0.0169	1.15
	HSPB7	0.0144	1.16
	ZNF385B	0.0308	1.16
	ACTB	0.0475	1.17
	KANK2	0.0114	1.17
	LPIN1	0.0375	1.17
	RAE1	0.0079	1.17
	OBSCN	0.0307	1.18
	MLLT6	0.0290	1.18
	CPXM1	0.0187	1.18
	TNFRSF18	0.0485	1.18
	TLE1	0.0436	1.18
	ZBTB7B	0.0020	1.19
	PRR5	0.0420	1.19
	NAV2	0.0067	1.19
	JDP2	0.0369	1.19

	SLC27A5	0.0355	1.22
	TGIF2	0.0410	1.23
	MARVELD1	0.0306	1.23
	HBA1	0.0388	1.23
	LIMK1	0.0182	1.23
	TSPYL2	0.0301	1.24
	C11orf84	0.0351	1.24
	CSNK1G2	0.0007	1.24
	ABCD1	0.0326	1.25
	ATXN7L3	0.0055	1.25
	CDV3	0.0193	1.25
	MAPKAPK2	0.0488	1.25
	LPPR3	0.0159	1.25
	RGS19	0.0320	1.26
	SQSTM1	0.0138	1.26
	THRA	0.0434	1.27
	HS1BP3	0.0183	1.27
	SLC1A5	0.0427	1.28
	C17orf70	0.0423	1.28
	SLC25A22	0.0306	1.29
	ARHGEF17	0.0011	1.29
	COL4A2	0.0181	1.30
	NMT1	0.0340	1.30
	EPHB4	0.0392	1.31
	RAB4B	0.0181	1.33
	COMMD4	0.0076	1.33
	GABARAP	0.0010	1.33
	RGS3	0.0088	1.33
	PRMT2	0.0477	1.33
	GTF2IRD1	0.0087	1.34
	COMT	0.0380	1.35
	PRDX5	0.0174	1.36
	ABL2	0.0475	1.37
	CSRP1	0.0005	1.37
	FAM78A	0.0069	1.37
	VPS53	0.0248	1.37
	CRELD2	0.0444	1.38
	ARAP3	0.0055	1.38
	UBL7	0.0282	1.38
	UBTF	0.0447	1.38
	SHC1	0.0261	1.39
	TENC1	0.0080	1.39
	MTA1	0.0080	1.39
	ZC3H7B	0.0336	1.40
	SLC9A1	0.0130	1.41

	CIC	0.0030	1.41
	ABHD14A	0.0085	1.41
	FSD1	0.0175	1.42
	EIF4G1	0.0336	1.43
	ACIN1	0.0030	1.43
	BCAR1	0.0141	1.44
	TUBB	0.0008	1.46
	UBE2J2	0.0428	1.46
	OGFR	0.0367	1.46
	TPI1	0.0385	1.46
	CDC25B	0.0189	1.47
	SMARCA4	0.0130	1.47
	TSPAN4	0.0444	1.47
	PIM3	0.0203	1.48
	CRAT	0.0067	1.49
	STAT6	0.0036	1.49
	TCF3	0.0182	1.49
	PILRB	0.0348	1.50
	DYNC1H1	0.0228	1.51
	AES	0.0063	1.51
	ELF4	0.0121	1.53
	MMP2	0.0028	1.54
	RAB5C	0.0010	1.55
	ACADVL	0.0202	1.55
	PCBP4	0.0475	1.56
	HMGA1	0.0167	1.57
	ZZEF1	0.0452	1.57
	ATP13A1	0.0039	1.57
	HES4	0.0496	1.57
	SCARA3	0.0210	1.58
	SLC29A1	0.0303	1.59
	CPNE1	0.0157	1.59
	RAVER1	0.0239	1.59
	PPP2R4	0.0220	1.63
	UCKL1	0.0427	1.64
	FBLIM1	0.0327	1.64
	CITED2	0.0364	1.64
	LRFN4	0.0386	1.64
	FSTL3	0.0214	1.65
	MARCKSL1	0.0454	1.65
	SGSH	0.0274	1.67
	MCM7	0.0417	1.67
	HSF1	0.0433	1.68
	PRPF31	0.0406	1.68
	ASMTL	0.0059	1.68

	HDAC7	0.0140	1.68
	DHCR7	0.0033	1.69
	MDK	0.0242	1.69
	PPP1R14B	0.0165	1.69
	PFN1	0.0154	1.69
	GNA11	0.0031	1.70
	SLC6A8	0.0150	1.70
	HOMER3	0.0009	1.70
	HIP1R	0.0280	1.73
	DBN1	0.0097	1.73
	MBOAT7	0.0231	1.74
	STUB1	0.0275	1.76
	CTSD	0.0072	1.77
	ECM1	0.0355	1.78
	FAM65A	0.0014	1.78
	SH2D3C	0.0316	1.78
	PLEC	0.0073	1.79
	FLOT1	0.0103	1.81
	RPP25	0.0192	1.85
	ACTR1A	0.0214	1.87
	LRP5	0.0194	1.92
	MYL9	0.0053	1.94
	LMNA	0.0057	1.96
	ZNF618	0.0034	2.06
	NT5DC2	0.0023	2.08
	PRKCSH	0.0137	2.11
	ARHGDIA	0.0058	2.15
	POLR2L	0.0119	2.15
	ZYX	0.0032	2.21
	CAMK2N1	0.0406	2.22
	MAZ	0.0010	2.25
	TGFB1I1	0.0056	2.33
	LTBP3	0.0070	2.35
	FLNA	0.0007	2.67
**REPIN1_GENES**	FAM76B	0.0239	-5.94
	EME1	0.0252	-3.19
	OGT	0.0258	-2.86
	FLCN	0.0303	-2.41
	RPS27L	0.0181	-2.36
	TMEFF2	0.0081	-1.96
	FOSL2	0.0106	-1.93
	MRPS25	0.0117	-1.92
	SLMO2	0.0447	-1.87
	FAM134A	0.0045	-1.86
	PDGFA	0.0087	-1.82

	TAF9B	0.0271	-1.74
	SCAF11	0.0258	-1.73
	KPNA3	0.0268	-1.70
	RAB14	0.0498	-1.69
	NEK1	0.0383	-1.67
	ATXN7	0.0420	-1.63
	FNIP1	0.0199	-1.63
	PRICKLE1	0.0263	-1.63
	PHAX	0.0070	-1.56
	ANKH	0.0028	-1.52
	NDUFS1	0.0341	-1.52
	HIPK2	0.0278	-1.48
	FOXN2	0.0463	-1.48
	TRAPPC2	0.0388	-1.48
	PLEKHA3	0.0219	-1.47
	PPP1R2	0.0340	-1.46
	MAP4K4	0.0201	-1.46
	HELZ	0.0259	-1.41
	KIAA0232	0.0058	-1.38
	RBM39	0.0239	-1.38
	PICALM	0.0440	-1.37
	CCNC	0.0369	-1.37
	METTL4	0.0364	-1.37
	GNB4	0.0275	-1.36
	DHRS12	0.0378	-1.36
	GUK1	0.0102	-1.33
	DDX3X	0.0261	-1.32
	NCS1	0.0330	-1.32
	PTPRE	0.0279	-1.32
	PTPRB	0.0486	-1.29
	ATF1	0.0250	-1.29
	SMAD2	0.0396	-1.28
	ARHGEF40	0.0339	-1.28
	PDGFC	0.0464	-1.27
	KARS	0.0052	-1.27
	NUP50	0.0184	-1.26
	ATP5F1	0.0026	-1.26
	RGS10	0.0178	-1.26
	LUC7L3	0.0066	-1.25
	CHCHD7	0.0473	-1.25
	B3GALNT2	0.0114	-1.24
	CMIP	0.0350	-1.23
	SLC10A7	0.0308	-1.23
	ZNF248	0.0221	-1.22
	DPP6	0.0283	-1.22

	CFLAR	0.0021	-1.21
	SNAP25	0.0336	-1.20
	SFT2D1	0.0115	-1.20
	MYLIP	0.0413	-1.20
	FBXL13	0.0039	-1.20
	RNF138	0.0345	-1.18
	HOPX	0.0278	-1.17
	SERP2	0.0346	-1.16
	MATR3	0.0300	-1.16
	BMP2K	0.0490	-1.16
	PKP4	0.0290	-1.16
	PKNOX1	0.0464	-1.14
	ZIC4	0.0051	-1.14
	SMU1	0.0362	-1.13
	SYT3	0.0493	-1.12
	EIF5	0.0467	-1.12
	SNTB1	0.0400	-1.11
	CASP9	0.0235	-1.10
	RPL22	0.0476	-1.10
	HNRNPAB	0.0310	-1.10
	ZIC1	0.0407	-1.07
	CASZ1	0.0483	1.07
	PPIA	0.0170	1.07
	GFRA1	0.0026	1.10
	CDHR5	0.0418	1.10
	RPS28	0.0117	1.10
	ANKRD9	0.0093	1.11
	RASGRP1	0.0221	1.11
	PAWR	0.0402	1.12
	MLX	0.0463	1.12
	UFD1L	0.0349	1.12
	BRF1	0.0412	1.12
	RARA	0.0020	1.13
	FOXH1	0.0376	1.14
	C17orf100	0.0432	1.14
	ATRNL1	0.0271	1.14
	SLC35C2	0.0299	1.14
	EPHB2	0.0169	1.15
	ZNF385B	0.0308	1.16
	SYNE1	0.0422	1.17
	TMEM37	0.0498	1.17
	HSP90AB1	0.0305	1.17
	HEY2	0.0357	1.18
	EPHA6	0.0237	1.18
	ZBTB7B	0.0020	1.19

	NAV2	0.0067	1.19
	MAPK8	0.0134	1.19
	FOXC2	0.0087	1.19
	CDH5	0.0311	1.20
	RASSF7	0.0241	1.23
	LIMK1	0.0182	1.23
	CSNK1G2	0.0007	1.24
	USF2	0.0105	1.25
	ATXN7L3	0.0055	1.25
	CDV3	0.0193	1.25
	BTBD6	0.0447	1.26
	CYB5R3	0.0141	1.26
	KCTD17	0.0386	1.27
	WIPI2	0.0071	1.28
	LRRC32	0.0370	1.28
	ZNF76	0.0481	1.28
	GIT1	0.0338	1.29
	SLC25A22	0.0306	1.29
	JMJD8	0.0002	1.29
	COL4A2	0.0181	1.30
	LTBP2	0.0101	1.30
	EPHB4	0.0392	1.31
	KIAA0319L	0.0053	1.31
	TSNARE1	0.0377	1.31
	COMMD5	0.0068	1.31
	CLCN7	0.0460	1.31
	CAV1	0.0497	1.33
	RGS3	0.0088	1.33
	GANAB	0.0341	1.33
	MADD	0.0418	1.33
	ARMC4	0.0205	1.34
	IFITM2	0.0224	1.35
	SEMA3F	0.0125	1.35
	TMEM141	0.0008	1.35
	ADAM15	0.0389	1.35
	CPNE2	0.0014	1.35
	GRK5	0.0292	1.36
	PRDX5	0.0174	1.36
	TONSL	0.0202	1.37
	BMP1	0.0049	1.37
	INO80B	0.0177	1.37
	FAM78A	0.0069	1.37
	VPS53	0.0248	1.37
	MAP1LC3A	0.0459	1.38
	TENC1	0.0080	1.39

	GP1BB	0.0125	1.40
	SLC9A1	0.0130	1.41
	CIC	0.0030	1.41
	GPR108	0.0479	1.41
	ILK	0.0046	1.42
	FAM127A	0.0206	1.43
	YBEY	0.0195	1.43
	EIF4G1	0.0336	1.43
	CTNNBIP1	0.0434	1.44
	PLD2	0.0011	1.44
	NCAPH2	0.0477	1.44
	TMEM8A	0.0088	1.45
	CAPNS1	0.0460	1.46
	RBPMS	0.0375	1.46
	MED25	0.0069	1.46
	UBAP2L	0.0333	1.47
	SMARCA4	0.0130	1.47
	AHCY	0.0095	1.47
	PIM3	0.0203	1.48
	SH2B1	0.0346	1.49
	CRAT	0.0067	1.49
	HSPG2	0.0028	1.49
	YIF1A	0.0343	1.51
	SYVN1	0.0310	1.52
	ADD1	0.0044	1.52
	CLTB	0.0220	1.53
	LZTS2	0.0230	1.55
	PCBP4	0.0475	1.56
	VPS28	0.0142	1.57
	ACVRL1	0.0414	1.57
	FARP1	0.0474	1.58
	SCARA3	0.0210	1.58
	GPS1	0.0466	1.58
	SLC29A1	0.0303	1.59
	RAVER1	0.0239	1.59
	PPDPF	0.0380	1.60
	FSCN1	0.0037	1.60
	CDK2AP2	0.0045	1.61
	UBTD1	0.0247	1.61
	POLR2E	0.0099	1.61
	RAB1B	0.0386	1.62
	CTDNEP1	0.0490	1.63
	TNK2	0.0261	1.63
	WBP2	0.0260	1.64
	JAG2	0.0435	1.64

	LRFN4	0.0386	1.64
	FSTL3	0.0214	1.65
	OBSL1	0.0398	1.65
	ASMTL	0.0059	1.68
	HDAC7	0.0140	1.68
	SLC25A39	0.0057	1.68
	MDK	0.0242	1.69
	SELO	0.0343	1.69
	PNKD	0.0317	1.71
	CABIN1	0.0224	1.71
	ARPC4	0.0444	1.73
	FGFR1	0.0061	1.73
	HGSNAT	0.0184	1.76
	MYO1D	0.0345	1.77
	PUF60	0.0129	1.78
	STRN4	0.0425	1.78
	TRIM47	0.0048	1.81
	CPSF1	0.0366	1.82
	GPR56	0.0364	1.83
	FAM129B	0.0320	1.84
	SDF4	0.0223	1.85
	RPP25	0.0192	1.85
	FASN	0.0263	1.87
	LYL1	0.0356	1.88
	PRKD2	0.0220	1.89
	ITGA5	0.0058	1.90
	LRP5	0.0194	1.92
	FXYD5	0.0020	1.93
	ISYNA1	0.0223	1.93
	ARF3	0.0182	1.94
	SPHK1	0.0440	1.96
	ACTN4	0.0087	1.98
	HGS	0.0136	1.98
	TLE2	0.0109	1.99
	DPYSL4	0.0499	2.06
	FOXP4	0.0472	2.11
	ARHGDIA	0.0058	2.15
	COL18A1	0.0157	2.17
	MAZ	0.0010	2.25
	ATP5D	0.0118	2.51
